# Estimation of Quasi-Stiffness of the Human Hip in the Stance Phase of Walking

**DOI:** 10.1371/journal.pone.0081841

**Published:** 2013-12-09

**Authors:** Kamran Shamaei, Gregory S. Sawicki, Aaron M. Dollar

**Affiliations:** 1 School of Engineering and Applied Science, Department of Mechanical Engineering and Materials Science, Yale University, New Haven, Connecticut, United States of America; 2 Joint Department of Biomedical Engineering, North Carolina State University and University of North Carolina at Chapel Hill, Raleigh, North Carolina, United States of America; University of California Davis, United States of America

## Abstract

This work presents a framework for selection of subject-specific quasi-stiffness of hip orthoses and exoskeletons, and other devices that are intended to emulate the biological performance of this joint during walking. The hip joint exhibits linear moment-angular excursion behavior in both the extension and flexion stages of the resilient loading-unloading phase that consists of terminal stance and initial swing phases. Here, we establish statistical models that can closely estimate the slope of linear fits to the moment-angle graph of the hip in this phase, termed as the quasi-stiffness of the hip. Employing an inverse dynamics analysis, we identify a series of parameters that can capture the nearly linear hip quasi-stiffnesses in the resilient loading phase. We then employ regression analysis on experimental moment-angle data of 216 gait trials across 26 human adults walking over a wide range of gait speeds (*0.75–2.63 m/s*) to obtain a set of general-form statistical models that estimate the hip quasi-stiffnesses using body weight and height, gait speed, and hip excursion. We show that the general-form models can closely estimate the hip quasi-stiffness in the extension (*R^2^ = 92%*) and flexion portions (*R^2^ = 89%*) of the resilient loading phase of the gait. We further simplify the general-form models and present a set of stature-based models that can estimate the hip quasi-stiffness for the preferred gait speed using only body weight and height with an average error of *27%* for the extension stage and *37%* for the flexion stage.

## Introduction

A number of engineered locomotion systems aim to emulate the biomechanical behavior of humans including anthropomorphic bipedal robots [1], [2], lower-limb wearable exoskeletons [3]–[6], and biologically-inspired prosthetic limbs [7]–[10]. Robust performance of these systems can be achieved using mechanisms that function similar to the biological joints. These mechanisms should ideally be built upon a foundation of simple models (theoretical or empirical) that can accurately characterize the normal mechanical behavior of the human joints during the locomotion tasks [11]–[13]. Therefore, design of these locomotion systems requires knowledge of how individual joints behave during locomotion tasks. To this end, researchers have used both empirical and theoretical approaches to characterize human locomotion. Experiments have been performed to measure the kinetics and kinematics of the human joints in locomotion tasks using gait laboratory equipment [14]–[16], and whole-leg models have been implemented with a range of complexity that can generate human locomotion patterns [1], [13], [17]–[25]. Researchers have also investigated the torque generation capabilities of the joints in terms of the passive and active stiffness using system identification techniques that employ statistical analyses and experimental data [26]–[28]. Most of these studies examined the joint and leg stiffness under controlled conditions and in specific tasks such as hopping or lateral balance; making it difficult to extend results to the behavior of joints during walking/running [21], [26], [27], [29]–[31]. However, a common finding from all of these approaches is that compliance (i.e. springy limb behavior) plays a central role in shaping human motion.

Previous studies show that the lower extremity joints have moment-angle patterns with highly linear phases during gait, especially during periods of high loading [32]–[36]. These findings have motivated incorporation of passive elastic components in the design of lower extremity orthoses/exoskeletons and prostheses to unload/mimic the musculature system function [37]–[39]. Moreover, the loading/unloading behavior of the lower extremity joints has been investigated using the concept of quasi-stiffness or “dynamic stiffness” [32]–[36], [40]–[45]. The quasi-stiffness is defined as the slope of the linear fit to the moment-angle curve of a joint in a specific task. One should note that the quasi-stiffness is usually defined for the overall performance of a joint in a gait task wherein the joint shows linear moment-angle behavior; hence, it should be distinguished from the passive and active stiffness of a joint defined as a specific function of angle and time [26], [27], [46]. The concept of quasi-stiffness applies particularly well to major loading phases of the lower extremity joints, mainly the ankle joint during stance phase, knee during the weight acceptance phase, and hip joint during the late stance and early swing phase of walking [32]–[36], [41]. From a design standpoint, a spring with a rotational stiffness equal to the joint quasi-stiffness can closely mimic the function of that joint in that specific task. Accordingly, many researchers develop and size prostheses according to the *average* joint quasi-stiffness (and additional tuning on the user) [9], [32]–[34], [40]–[45]. Our previous research shows that the quasi-stiffness of lower limb joints can substantially change according to the gait conditions and subject size [33]–[36]. Moreover, a simple and fast measurement of the joint quasi-stiffness for patients in a gait laboratory is very difficult. Therefore, the design of prostheses and orthoses could benefit from subject and gait specific model estimates for the quasi-stiffness of lower extremity joints in the key loading/unloading phases of gait.

The overall goal of this work was to establish statistical models that can closely characterize the hip quasi-stiffness in the late stance and early swing phases (i.e. resilient loading phase) of walking for adult humans spanning body size (height and weight) across a wide range of walking speeds. This work is an extension of our previous efforts to characterize the quasi-stiffness of other lower extremity joints during the major loading phases of the gait and establish predictive models that promise to aid in the development of biologically-inspired assistive devices (e.g. exoskeletons, orthoses, and prostheses) [4], [33]–[38], [47]. Generalized biomechanical models that can explain subject-specific variability of the behavior of the hip joint could allow the stiffness of the hip joint of a device to be selected in advance and according to the gait requirements of the user.

We begin with a description of the hip moment-angle behavior, modeling approach and data collection methods used in the study. To extract the models, we obtain a generic equation for the hip moment through an inverse dynamics analysis and identify a subset of factors that can explain the quasi-stiffnesses of the hip during stance phase. We then employ a data set including the moment-angle information for 216 gait trials across 26 human adult subjects spanning a substantial range of body sizes and gait speeds to extract the coefficients of each factor and obtain a series of *general-form* statistical models. We show that the models can closely estimate the hip quasi-stiffnesses across the gait trials examined.

The general-form models estimate the hip quasi-stiffness using the magnitude of hip excursion, gait speed, and body size. For design occasions where it would be undesirably time-consuming or expensive or where hip kinematics cannot be easily and repeatedly characterized (e.g. in an orthosis for a spinal cord injury patient), we develop more simplified models that only include the body size. These models favor the design of compliant assistive devices that are versatile enough to perform well over a range of speeds around the preferred gait speed. These simplified equations are termed *stature-based* models, and are only functions of body weight and height.

## Methods

### Hip Phases of Motion in a Gait Cycle and Terminology

**Figure 1:**
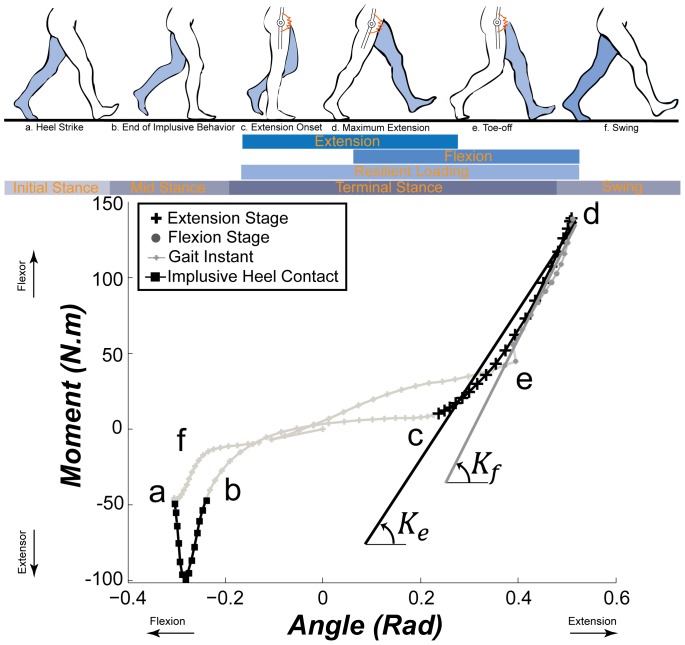
Top: Schematic poses of the human leg in different phases of a walking stride (timing is adapted from [76]), Bottom: Hip moment-angle graph for a representative subject walking at *2.17 m/s*. Letters *a*–*f* on the graph correspond to the poses shown during a typical walking cycle. Quasi-stiffnesses of the hip in the resilient loading phase are defined as the slopes of the best linear fits to the moment-angle curve of *c*–*d* in the extension stage, and *d*–*e* in the flexion stage.

The human hip is primarily involved in stabilizing the trunk and driving/braking the thigh and experiences two arcs of motion in a gait cycle: an extension and a flexion arc [14], [48], [49]. The stance phase of a gait cycle in the sagittal plane can be divided into initial, mid, and terminal stance sub-phases (schematically shown in Fig 1, top). In the initial stance phase, the direction of the ground reaction force anterior to the center of the hip in combination with its large moment arm induces an impulsive net extensor muscle moment on the hip joint and forms a spike-shape in the moment-angle graph (Fig 1, *a*–*b*). In the mid stance phase, the hip net extensor moment shows a moderate increase in the sagittal plane (Fig. 1, *b*–*c*). In the terminal stance, the hip undergoes a *resilient loading* phase that is composed of an extension stage where the energy is stored (i.e. net flexor moment and extensor angular velocity), and a flexion stage where the stored energy is released (i.e. net flexor moment and flexor angular velocity). This primary loading-unloading phase of the hip begins around the onset of the terminal stance and extends into the initial swing phase with high loading that is concurrent with a phase of storage and then return of mechanical energy. In this work, we characterize the moment-angle behavior of the hip in this resilient loading phase of the gait (Fig. 1, *c*–*e*), where the hip exhibits a nearly linear extension stage (*c*–*d*) and flexion stages (*d*–*e*) [32]. We focus on this phase because the high loading requirements of the hip joint during this time make it likely that loss of normal function of the musculoskeletal system (e.g. in patients with spinal cord injury and stroke) could significantly disrupt normal hip mechanical function. In addition, the mechanical behavior during the resilient loading phase indicates an ideal opportunity for ‘spring-like’ external assistance at the hip joint (Fig. 1, *c*–*e*) [38].

We investigated the hip behavior in each stage using the slope of a linear fit to the moment-angle graph in that stage and name it the quasi-stiffness of that particular stage. For example, we fit a line on the moment-angle data between point *c* and *d* to obtain the quasi-stiffness of the extension stage (*K_e_*), and between *d* and *e* for the flexion stage (*K_f_*) of the resilient loading phase of the hip (see Fig. 1, bottom). The excursion of the hip joint in the extension stage (*θ_e_*) was obtained by subtracting the initial from the final hip angle in the sagittal plane during the extension stage. Similarly, the hip excursion in the flexion stage (*θ_f_*) was calculated as the difference between the hip angle in the sagittal plane at the beginning and end of the flexion stage.

### Identifying the Model Parameters and Form of Fits

We identified the model parameters using a generic equation for the hip joint moment obtained through an inverse dynamics analysis, as documented in Appendix S1, Fig. S1 and Table S1. We simplified this generic equation for the instant of hip maximum extension (Fig. 1, point *d*) and extracted an equation for the hip moment on the sagittal plane (*X*–*Y* of Fig. S1). Next, we investigated the terms of the simplified equation and correlated them with the body and gait parameters. The body parameters include weight (*W*) and height (*H*), and the gait parameters include gait speed (*V*) and joint excursions (*θ_e_* and *θ_f_*). Considering that the hip behaves linearly in the extension and flexion stages of the resilient loading phase, we found a correlation between the hip quasi-stiffnesses (*K_e_* and *K_f_*) and the body and gait parameters.

The generic equation of the hip moment was obtained as (refer to Appendix S1 and Table S1 for the definition of each parameter that follows):
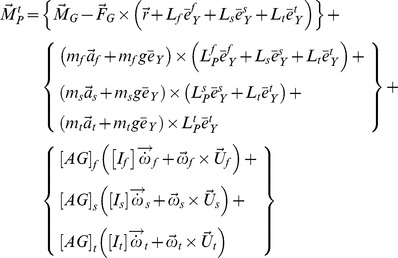
(1)


To simplify equation (1) for the instant of maximum extension, we applied a series of approximations. At this instant of the stride, the support leg is instantaneously nearly stationary (i.e. 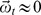, 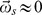 and 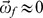). The effect of linear and angular acceleration of the support leg segments is also negligible when compared with the acceleration of the rest of the body (i.e. 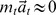 and 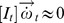, 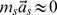 and 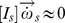, and 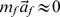 and 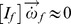). Similarly, the effect of the weight of the support limbs can be neglected (i.e. 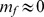, 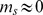, and 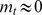) when compared to those of the rest of the body. We applied these assumptions in the generic equation (1) and obtained the following simplified equation for the hip moment at the instant of maximum extension:
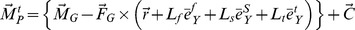
(2)where, 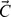 is a constant vector and reflects the effect of the neglected terms. We obtained the sagittal-plane component of the hip moment at the instant of maximum extension (point *d* in Fig.1) from equation (2) as:
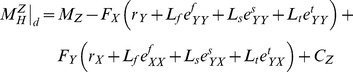
(3)where, 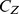 is the *Z*-component of 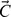. Moreover, 
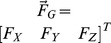
, 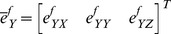, 
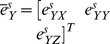
, and 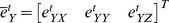, and 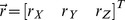, as explained in the Appendix and Table S1. At the instant of maximum extension, the foot is instantaneously stationary, and the toe is on while the heel is off the ground. Thus, 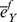 was assumed to be an instantaneously constant vector that makes an angle (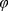) with the *X*-axis. Previous research shows that the center of pressure (*COP*, shown in Fig. S1) at this time lies around the heads of the metatarsi and end of the toe [48], [50]. 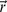 connects the *COP* to the tip of toe; hence, 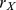 would be correlated with the toe length, and 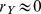. Since the toe length is proportional to the body height, 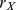 would also be correlated with  [51]. At the instant of maximum extension in the terminal stance phase, the shank and thigh segments are approximately aligned and make an angle with the vertical line that is equal to the hip angle (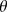). Therefore, we assumed 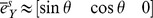 and 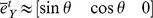, assuming that the leg moves only on the sagittal plane. Considering that the hip angle is small during normal walking we assumed 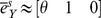 and 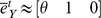. Including the aforementioned assumptions, we obtained:



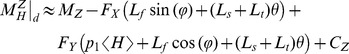
(4)


In this text, 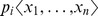 denotes an arbitrary first-order polynomial of 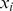
's in its general form. The ground reaction moment can be neglected when compared with the substantial hip moment at this instant (i.e. 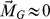). When the hip is maximally extended, the ground reaction force (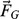) shows a local maximum. The extrema of normalized ground reaction force (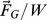) are linearly correlated with the gait speed [52]:
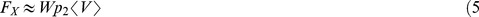
(5-a)

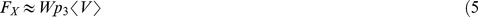
(5-b)


Moreover, investigation of the variability of foot kinematics shows that an increase in the gait speed leads to an increase in the hallux dorsi-flexion [53]. Accordingly, we approximated the foot angle with the ground (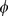, as shown in Fig. S1) by a linear function of the gait speed (i.e. 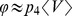). Implementing the aforementioned assumptions and equations (5-a and b) in equation (4) yielded:



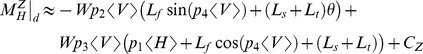
(6)


We approximated the trigonometric functions by the first two terms of their Taylor series expansions:



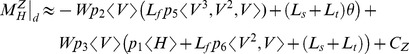
(7)


The hip angle (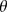) at the instant of maximum extension shows a linear correlation with the gait speed [54]. Furthermore, the anthropometric relationships suggest that 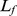, 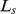, and 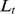 are all proportional to 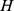 [51]. Therefore:



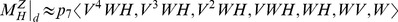
(8)


In the previous section, we explained that the hip behaves linearly in both extension and flexion stages of the resilient loading phase. Therefore:



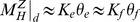
(9)


Equations (8) and (9) contain identical left hand sides. Therefore, we obtained the following forms for the models of the hip quasi-stiffness in the extension (*K_e_*) and flexion (*K_f_*) stages of the resilient loading phase of the hip:



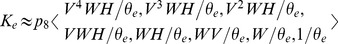
(10-a)




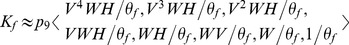
(10-b)


In other words, 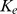 could be modeled by a first order polynomial of 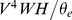, 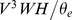, 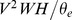, 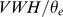, 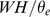, 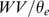, 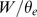, and 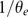 (and a function of only *V*, 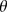, *H*, and *W*); and similarly for 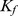 with 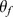 instead of 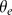.

### Experimental Protocol, Data Extraction and Statistical Analysis

Researchers from three different labs provided us with the hip moment and angle data, and the collection procedures for 216 trials across 26 healthy male and female adults with a reasonably wide range of mass (*46*–*94.0 kg*) and height (*1.43*–*1.87 m*). The trials included the preferred gait speed for subjects 15 to 26, and a wide range of gait speeds for subjects 1 to 14 (*0.75*–*2.63 m/s*). Data was compiled using:

1. Nine subjects (subjects 1 to 9 in Table 1) at Human PoWeR Lab, NC State University walking on a treadmill, as detailed in [16].
2. Five subjects (subjects 10 to 14 in Table 1) at Biomechanics Lab, East Carolina University walking on level ground. The general procedures used to obtain the ground reaction force, sagittal plane hip joint angular position and torque are described elsewhere [55]. We detail here the specific procedures relevant to the purpose of this study. All participants read and signed an informed consent form approved by the University Institutional Review Board at East Carolina University. Using a *15 m* walkway, force platform (AMTI, Watertown, MA) and eight camera motion capture system (Qualisys, Gothenberg, Sweden), three dimensional ground reaction force and linear position data describing the right lower extremity and pelvis were obtained from each participant during 20 walking trials of different velocities ranging from *1.01* to *2.63* *m/s*. Each participant was initially tested at a self-selected, moderate walking speed the mean of which was *1.63*±*0.03* *m/s*. Subsequently, the 19 remaining trials per participant were collected in an approximately random order of walking velocities. Participants were instructed to walk at various speeds with instructions such as, “walk at a moderately fast pace,” “walk at a very slow pace,” and “walk at your fastest pace.” The mean walking velocity for all trials was *1.77*±*0.36 m/s*. All participants had similar minimum and maximum walking velocities and therefore similar ranges of walking velocities. Additionally, the 20 walking velocities for each participant were moderately evenly distributed through the range of velocities from slowest to fastest velocities. Qualisys Track Manager and Visual 3D software (C-Motion, Gaithersburg, MD) were used to calculate the hip joint angular position and torque through the stance phase of walking in each trial from the linear position and ground reaction force data. The subject consents, collection protocols and data analysis for subject groups 1 and 2 are detailed elsewhere [16], [55].
3. Twelve subjects (subjects 15 to 26) at Laboratory of Biomedical Technologies at Politecnico Di Milano walking on level ground. For subject group 3, kinematic data were collected by using a motion analyzer (ELITe System, BTS, Italy) based on TV-signals processing [56]. Retro-reflective markers were positioned on the body according to a predefined protocol [57], [58]. Eight TV-cameras were located in the laboratory as to detect a calibrated volume *3 m* long, *2 m* high, *1.5 m* wide. Accuracy of the 3D coordinates was approximately *1 mm* in the calibrated volume; frequency of acquisition was *50 Hz*. Kinetic data were obtained by measuring ground reaction forces and moments through a dynamometric force platform (Kistler 9281B, Winterthur, Switzerland). Data processing to estimate joint centers and to compute joint moments, based on an inverse dynamics approach, has been described elsewhere [59], and was validated, more recently, in a comparative study performed [60]. All participants read and signed informed consent forms approved by the Institutional Review Boards of the universities where the experiments were conducted including: a. East Carolina University, b. North Carolina State University, and c. Politecnico Di Milano.

**Table 1:** Details on Subjects and Experimental Trials used for Regression Fits.

Subject	Gender	#Trial	*W*	*H*	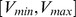	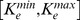	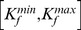	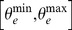	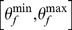	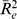	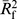
1‡	M	4	92.3	1.86	[0.75,2.00]	[95,405]	[160,401]	[8], [14]	[7], [16]	94	87
2‡	M	4	68.4	1.70	[0.75,2.00]	[91,184]	[88,248]	[11], [16]	[5], [15]	93	91
3‡	M	4	65.6	1.65	[0.75,2.00]	[116,187]	[112,318]	[10], [23]	[8], [12]	93	95
4‡	M	4	94.0	1.86	[0.75,2.00]	[166,326]	[126,507]	[9], [16]	[4], [31]	96	95
5‡	M	4	68.1	1.72	[0.75,2.00]	[155,342]	[196,423]	[10], [14]	[8], [10]	97	91
6‡	F	4	57.7	1.43	[0.75,2.00]	[134,226]	[109,266]	[10], [12]	[7], [13]	92	93
7‡	F	4	63.1	1.45	[0.75,2.00]	[152,467]	[143,632]	[6], [10]	[4], [10]	95	89
8‡	F	4	65.7	1.75	[0.75,2.00]	[95,290]	[125,427]	[11], [15]	[4], [9]	94	91
9‡	F	4	75.9	1.80	[0.75,2.00]	[141,1775]	[46,369]	[1], [14]	[2], [13]	97	91
10†	M	20	85.7	1.74	[1.26,2.43]	[404,972]	[632,1400]	[3], [16]	[2], [9]	99	87
11†	M	20	79.2	1.82	[1.38,2.25]	[199,358]	[149,489]	[7], [21]	[5], [15]	96	90
12†	M	20	62.1	1.64	[1.04,2.29]	[174,412]	[9,658]	[3], [18]	[1], [9]	98	67
13†	M	20	62.0	1.62	[1.01,2.44]	[324,577]	[22,312]	[2], [7]	[2], [14]	98	80
14†	M	20	75.1	1.77	[1.30,2.63]	[268,646]	[348,1032]	[3], [20]	[3], [10]	99	86
15•	F	5	58.0	1.60	[1.00,1.25]	[130,307]	[105,200]	[5], [14]	[9], [18]	98	91
16•	F	6	56.0	1.60	[1.18,1.26]	[130,255]	[82,171]	[6], [15]	[13], [24]	99	91
17•	F	9	48.0	1.58	[0.96,1.08]	[124,204]	[50,124]	[5], [16]	[9], [26]	99	90
18•	F	7	46.0	1.60	[1.08,1.19]	[112,173]	[32,85]	[6], [10]	[11], [18]	96	88
19•	F	4	53.0	1.61	[1.12,1.28]	[117,234]	[30,86]	[6], [22]	[11], [22]	93	95
20•	F	5	53.0	1.67	[1.3,1.34]	[165,499]	[44,143]	[1], [13]	[7], [13]	86	92
21•	M	7	90.0	1.80	[1.24,1.31]	[352,625]	[147,233]	[4], [8]	[13], [16]	98	95
22•	M	9	55.0	1.73	[1.18,1.26]	[153,291]	[44,107]	[4], [8]	[13], [33]	99	97
23•	M	5	77.0	1.80	[1.36,1.42]	[160,234]	[93,162]	[10], [23]	[15], [38]	97	93
24•	M	4	75.0	1.87	[1.39,1.48]	[246,492]	[208,305]	[7], [15]	[11], [15]	98	91
25•	M	6	71.0	1.72	[1.27,1.35]	[187,301]	[49,136]	[4], [13]	[12], [22]	84	93
26•	M	13	72.0	1.81	[1.13,1.27]	[227,599]	[50,158]	[2], [9]	[8], [29]	97	92
		**Mean**	**69.1**	**1.71**	**1.51**	**320**	**335**	**9.3**	**10.3**	**97**	**87**
		**SD**	**12.4**	**0.10**	**0.41**	**196**	**329**	**4.8**	**6.5**	**5**	**13**

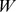
: Body weight (kg), and 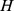: Body height (m),

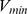
 and 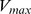: Minimum and maximum gait speed (m/s)

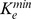
 and 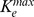: Minimum and maximum hip quasi-stiffness in extension stage (Nm/rad)

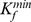
 and 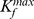: Minimum and maximum hip quasi-stiffness in flexion stage (Nm/rad)

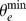
 and 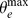 : Minimum and maximum hip excursion in extension stage (deg)

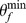
 and 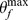 : Minimum and maximum hip excursion in flexion stage (deg)

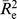
: Average 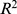 of the linear fit on moment-angle curve in extension stage

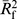
: Average 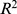 of the linear fit on moment-angle curve in flexion stage

‡Data collected at Human PoWeR Lab, NC State University [16]

†Data collected at Biomechanics Lab, East Carolina University [55]

•Data collected at Laboratory of Biomedical Technologies at Politecnico Di Milano.

We first plotted the moment-angle graphs for each trial (similar to Fig. 1-bottom), and identified the onset of the extension stage as the point of local deflection (i.e. where 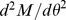
showed a local discontinuity and theoretically where 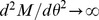) in the moment-angle graph of the hip in terminal stance (point *c*) and the end of flexion stage as the point of local discontinuity in the moment-angle graph of the hip in the initial swing phase (point *e*). This corresponds quite closely to the point where the mechanical power curve of the hip crosses zero (∼25% of the gait cycle), and then the hip exhibits power absorption followed by generation (i.e. a stretch shortening cycle) [16]. The top right corner of the moment-angle curve (i.e. point of maximum extension) was selected as the instant of maximum extension (point *d*) and is where the hip transfers from extension to flexion. Accordingly, the data points between *c* and *d* compose the extension stage and between *d* and *e* the flexion stage. We fitted a line to the data points of each stage using Least Square Regression method. We obtained *K_e_* and *K_f_* as the slope of the fitted lines of the extension and flexion stages (as described in the previous section).

In the previous section, we found that *K_e_* and *K_f_* could be modeled by a series of parameters that are collinear. Therefore, we applied stratified cross-validation (CV) on the model structure by removing one subject at a time and applying Partial Least Square (PLS) analysis. This allowed us to investigate the predictive ability of the predictors and to find the optimal number of components that could best describe the response variables (i.e. quasi-stiffnesses, *K_e_* and *K_f_*) [61]–[63]. The first order polynomials of equations 10-a and b led us to apply first-order linear regression between the values of *K_e_* and *K_f_*, and the parameters that these equations suggested. In this case, we used Least Square Regression because the predictor parameters were known (i.e. accurately measured). We started with a linear regression including all of the predictors of equations 10-a and b. Then, we used a step-wise regression and iteratively removed the non-significant terms (*p>0.05*) of the regressed polynomials until we obtained polynomials that only included significant terms. These polynomials were termed as the *general-form models*.

### Stature-Based Models

To obtain more simplified models that only included the body stature (*W* and *H*), we simplified the general-form models for the preferred gait speed. Since the subjects had different body sizes, we used the non-dimensional speed (Froude number: *Fr = V^2^/gl,* where *l* is the leg length and *g* is the acceleration due to gravity) instead of the actual gait speed. *Fr = 0.25* is an acceptable estimate of the preferred gait speed for subjects with different body size [64]–[67]. Assuming an anthropometric relationship of *l = 0.491 H* [51], the optimal or “preferred” gait speed was approximated as:
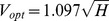
(9)


We observed that the general-form models mildly depended on the hip excursion, and the hip excursions demonstrate low variability around the optimal gait speed of *Fr = 0.25* (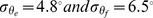). Therefore, we simplified the general models by merely substituting the hip excursions with the mean values of 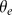 and 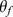 around the optimal gait speed (i.e. 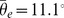
and 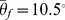), and the gait speed into equation (9). We termed the resultant equations as the *stature-based models,* intended to estimate the quasi-stiffnesses (*K_e_* and *K_f_*) of the hip at the preferred gait speed only as functions of *H* and *W*.

## Results

The linear fits (similar to that shown in Fig. 1-bottom) on the moment-angle graph of the hip joint in the extension stage showed an average *R^2^* of 97% in the extension and 87% in the flexion stages of the resilient loading phase (see Table 1). High *R^2^* values for nearly all subjects imply that the *hip behaves highly linearly in the extension and flexion stages of the resilient loading phase*. Table 1 also shows the range of hip quasi-stiffness and excursions across trials, and the average *R^2^* in the extension and flexion stages. *K_f_* showed higher variability (standard deviation) compared with *K_e_*. For the gait trials examined here, *K_e_* ranged from a minimum value of *91 Nm/rad* for subject 2 walking at *0.75 m/s* to a maximum value of *1775 Nm/rad* for subject 9 walking at *1.75 m/s*. *K_f_* ranged from a minimum value of *9 Nm/rad* for subject 12 walking at *1.12 m/s* to a maximum value of *1400 Nm/rad* for subject 10 walking at *1.65 m/s*. The average values of 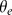 and 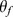
over all gait trials were calculated as *9.3*° and *10.3*°, respectively. The average values of 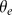 and 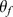 for the trials with gait speeds closest to *Fr = 0.25* were calculated as *11.1*° and *10.5*°. We also examined the linearity of the moment-angle performance of the hip joint over the rest of the gait cycle. In contrast to the findings of other researchers [32], we did not observe consistent linear behavior in initial and mid stance phase, and the rest of the swing phase (i.e. Fig. 1, b–c and e–f).

The PLS-CV analysis results are outlined in Table 2 including the number of optimal components, *R^2^*, and predicted *R^2^*. The CV analysis showed that 7 and 8 components can optimally describe the response parameters *K_e_* and *K_f_*, respectively, and resulted in minimal predicted residual sums of squares (PRESS statistics evaluated on the removed subsets). High values of *R^2^* obtained in the PLS-CV analysis confirmed that the parameters identified through the inverse dynamics analysis have the ability to constitute predictive models for *K_e_* and *K_f_*.

**Table 2:** General-Form Models to Predict the Quasi-Stiffness of the Hip Joint in the Resilient Loading Phase of Normal Walking.

Phase	Model	Unit	Error	PLS-CV #Comp.	PLS-CV R^2^	PLS-CV Predicted R^2^	Fit Quality
Extension	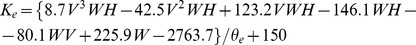	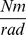	18%	7	91%	81%	R^2^ = 92%, p<0.0001
Flexion	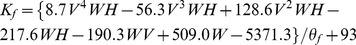	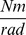	30%	8	89%	76%	R^2^ = 89%, p<0.0001

W: Weight (kg), H: Height (m), V: Gait Speed (m/s), and 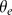 and 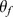 : Joint excursions (deg).

Table 2 also outlines the results of the Least Square Regression analyses including the general-form models of *K_e_* and *K_f_*, average error of the models, and fit quality. We found 6 and 5 gait trials respectively, wherein *K_e_* and *K_f_* showed outlier behavior for the predictions of the general-form models. The fit quality measures for the models of *K_e_* and *K_f_* were (*R^2^ = 92%, p<0.0001*) for *K_e_* and (*R^2^ = 89%, p<0.0001*) for *K_f_*, as reported in Table 2. The regression analysis also showed that the coefficients of the polynomials of the general-form models were significantly greater than zero (*p<0.0001* for the coefficients of the polynomials of *K_e_* and *K_f_*). We also observed that the residuals of the trials were normally distributed, and the order of data collection did not have any notable correlation with the residuals. This implies that the gait type (treadmill versus overground walking) did not have notable effect on the predictions of the models. We did however find slightly greater values for the residuals of the data of subjects 10 to 14 collected at East Carolina University.

**Figure 2:**
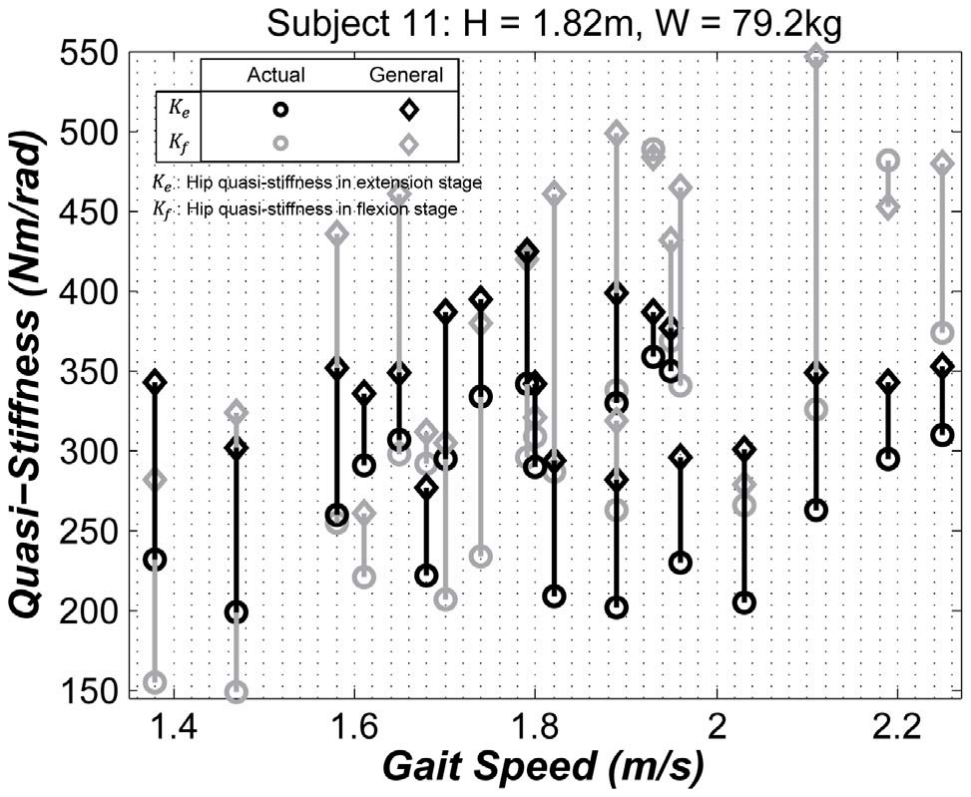
Hip quasi-stiffness for subject 11, as an example, in extension (black) and flexion (gray) stages plotted against the gait speed. The circles indicate the experimental values and the diamonds indicate the predictions of the general-form models listed in Table 2.

Fig. 2 shows the results of the predictions of the general-form models and the experimental values of *K_e_* and *K_f_* for a subject with a wide range of gait speeds *V = *[*1.38 m/s, 2.25 m/s*], and body weight *W = 79.2 Kg* and height *H = 1.82 m* close to the average for adults. We found that *K_e_* values can increase or decrease, whereas *K_f_* values moderately increase as the gait speed increases. We also observed that *K_e_* and *K_f_* were relatively close at moderate gait speeds, and diverged at low and high gait speeds. We observed similar quasi-stiffness dependence on gait speed for nearly all subjects 1 to 14 who walked at a wide range of speeds other than the preferred gait speed. Subject 10 showed outlier behavior, in that the range of *K_e_* values were substantially higher than *K_f_* values.

**Figure 3:**
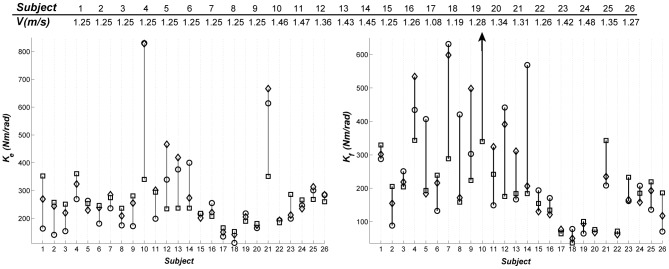
Left: The hip quasi-stiffness in the extension stage of the resilient loading phase of the gait. Right: The hip quasi-stiffness in the flexion stage of the resilient loading phase of the gait. The experimental values are shown by circles and the predictions of the general-form model by diamonds. The black arrow indicates the outlier behavior of subject 10 around the optimal gait speed.

Table 3 lists the stature-based models for *K_e_* and *K_f_*, average error of the models, and the assumptions that were made to obtain the models. We cannot report the *R^2^* for the model predictions because we do not have the actual values of the preferred gait speed for all of the subjects. Instead, we obtained the errors for the predictions of the stature-based models for the gait speeds that are closest to *Fr = 0.25*. Fig. 3 shows the predictions of the stature-based models for these gait trials. Subjects 10 and 21 exhibited outlier behavior (more than 50% error) for the stature-based models of *K_e_* and subjects 7, 10 and 14 exhibited outlier behavior for *K_f_* The stature-based models predicted *K_e_* and *K_f_* with average errors of 27% and 37% excluding the aforementioned outliers, and average errors of *33%* and *48%* when including them.

**Table 3:** Stature-Based Models to Predict the Quasi-Stiffness of the Hip Joint in the Resilient Loading Phase of Normal Walking at Optimal Gait Speed.

Phase	Model	Unit	Error	Conditions
Extension	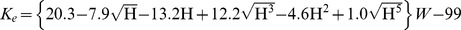	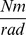	27%	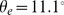 and 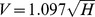
Flexion	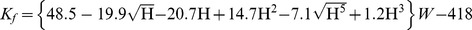	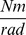	37%	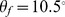 and 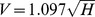

W: Weight (kg) and H: Height (m).

## Discussion

In this work, we established two statistical models that can closely estimate the quasi-stiffnesses of the hip in the resilient loading phase of walking (i.e. terminal stance phase through initial swing phase). To develop the models, we derived a generic equation for the hip moment through an inverse dynamics analysis, simplified it for the resilient loading phase, and obtained optimal parameters that can best describe the hip quasi-stiffnesses. We then used a data set spanning a relatively wide range of gait speeds and body sizes to construct the general-form statistical models through regression analysis. We found general-form models as functions of body weight and height, gait speed, and hip excursion. Additionally, we simplified the general-form models for the preferred gait speed and obtained more simplified models (i.e. design-oriented) that are only functions of body weight and height.

We observed very high *R^2^* values for the linear fits to moment-angle curves of the hip in the extension and flexion stages, implying a linear behavior of the hip in these stages (Fig. 1 *c*–*d* and *d*–*e*). This finding is in close agreement with the findings of other researchers [32]. Furthermore, we found that the slope of the linear fits in extension versus flexion tended to be closer to each other at low and high gait speeds (*spring-type behavior*). At moderate gait speeds, the hip behaved linearly but the slopes of the fitted lines in extension and flexion tended to deviate from one another. From a design point of view, the findings of this research suggest that devices including exoskeletons, prostheses, and biped robots could mimic the behavior of the hip joint in the resilient loading phase using a compliant spring, provided it behaves nearly linearly in extension and flexion stages of this phase. More specifically, the device design could employ a torsional spring stiffness sized based on the body size and gait speed to approximate the function of an unaffected hip joint in the resilient loading phase (Tables 2 and 3). We also note, that for the initial stance phase of walking, the spike-shape moment-angle graph of the hip suggests that a rigid locking mechanism might be most appropriate to emulate the hip joint mechanics.

Researchers in the field of exoskeletons have mostly focused on designing fully active hip joints with a range of sophistication [3], [68]–[72]. Considering the large moment requirement of the hip, these exoskeletons have remained bulky and heavy to carry. As a result, the current hip exoskeletons have primarily been tethered and used for rehabilitation purposes [69], [71], [72]. Recent approaches tend to employ more passive components such as springs in the design of these devices to realize a more streamlined form factor [4], [38], [47], [73]. Selection of the spring components of these quasi-passive devices requires *a priori* knowledge of the quasi-stiffness of the hip joint; knowledge that requires a time consuming gait lab analysis for each specific user. To date, most prostheses and orthoses/exoskeleton designers utilize the average quasi-stiffness extracted from the kinetic and kinematic data of sample healthy subjects [4], [7], [9], [20], [38], [47], [73]–[75]. For example, the designers of a quasi-passive exoskeleton used a spring with stiffness of ∼*112* *Nm/rad,* that was obtained from a linear fit to a sample gait cycle [4]. However, the sample populations are usually composed of individuals with weight, height, and preferred gait speed that are not necessarily representative of the target user. For these cases, we suggest using the models developed here for the selection of the device spring stiffness. To examine the improvement in accuracy of estimation of the quasi-stiffness using our models compared with models that merely use the average quasi-stiffnesses, we found the average values of *K_e_* and *K_f_* for the gait data utilized in our study and examined the error between the average quasi-stiffnesses and the true subject-specific quasi-stiffnesses. Table 4 compares the estimation errors of the general-form models, stature-based models, and average models that use the mean values of *K_e_* and *K_f_* (as reported in Table 1). The results show much larger errors when the average values are utilized than values obtained with our models. Therefore, we hypothesize that selection of the stiffness of exoskeletal devices based on the models presented here would result in a more natural and user/gait-adaptable performance, an idea that should be explored in future experimental studies of walking with hip exoskeletons spanning a range of stiffness values.

**Table 4:** Average Error Values for Different Models.

Parameter	General-Form	Stature-Based	Average Values
*K_e_*	18%	27%	46%
*K_f_*	30%	37%	219%

Our study had a number of methodological limitations worth addressing. Our goal was to compile a ‘large comprehensive data set’ to capture the variation in hip joint mechanical behavior during walking across subjects size and gait speed. Despite this goal we were only able to include a relatively modest number of gait trials (i.e. 216 gait trials across 26 adults). Therefore, our results represent a ‘first effort’ that should be added to using more and more data over time in order to gain more and more confidence in our model estimates. We caution that our results should only be generalized to the range of weight, height, and gait speed that the examined subjects represent and only to the level that the statistical significance supports.

A second potential limitation to our approach was that our experimental analyses extracted gait parameters independent of gender and whether the inverse dynamics data was acquired on a treadmill or during over ground walking. In addition, we included data from three different gait laboratories in an attempt to include the widest range of subject sizes and gait speeds possible. Although, in theory, a more comprehensive data set should be better able to capture the behavior over a wider range of humans, differences in gender and/or equipment and measurement protocols between labs could introduce variability not by factors we included in our models. To address this possibility, we examined the correlation between the residuals from the model fits and (1) the gender of the subjects, (2) the location of the laboratory data collection, and (3) whether data was acquired on a treadmill or overground. We found that although the data from East Carolina University demonstrated slightly higher residuals in the model fits than the other locations; the data from males versus females and treadmill versus overground walking showed no differences in the residuals of their model fits. Thus we felt confident that, as desired, the factors included in our model sufficiently capture variability due to subjects' height, weight and walking speed rather than other potential factors. As mentioned earlier, ideally future studies should aim to generate highly controlled data sets from a single laboratory on many more individuals spanning larger ranges of body size and including equal numbers of males and females to gain additional confidence in the statistical estimates presented in this study.

A third limitation was that we had to apply several simplification steps in order to reduce detailed inverse dynamics equations for the hip moment to obtain the minimal forms for the relationships describing quasi-stiffness in the sagittal plane as a function of stature and speed. These simplifications likely introduced small errors worth noting. For example, the eliminated terms of the generic equation for the hip moment could have introduced additional linear and non-linear predictors other than what equations (10-a) and (10-b) suggest. To check whether this was the case, we investigated additional potential linear forms and predictors (e.g. 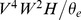 and 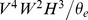
and etc.) capable of capturing the effect of the eliminated terms of the generic equation of the hip moment, and found that these additions were insignificant and resulted in no notable improvement in the models in terms of *R^2^* values and magnitude of residuals.

Finally, we only investigated the behavior of the hip in the resilient loading phase for walking on level ground. We chose this period because it is when the sagittal plane moments reached their highest peak values and exhibited nearly linear loading/unloading behavior (i.e. ‘spring-like’ mechanics). This phase is also the time during the stride when passive spring loaded assistive devices would likely be most effective. Future work could extend this approach to characterize hip quasi-stiffness during additional locomotion behaviors including running and walking on rough terrain, sloped ground, with load carriage. It should also be possible to reformulate the generic inverse dynamics equations for application in different gait phases and to estimate additional gait parameters (e.g. hip joint net work). Using similar statistical approaches to those presented here, it would also be interesting to characterize hip joint mechanics in other clinical populations (e.g. pediatric cerebral palsy, spinal cord injury, stroke, and aging).

In summary, the findings of this research suggest that designers could employ more passive components (i.e. springs) in the design of hip exoskeletal devices intended to reduce biological muscle forces during the terminal stance and initial swing phases of walking. We observed that the resilient loading phase typically starts when the hip is extended to ∼10° with respect to the standing configuration. This means a purely passive hip exoskeleton using a spring to produce flexion torque could be employed with a slack length/angle that causes it to begin storing elastic energy as the hip extends at the onset of the resilient phase and then later release that energy to help flex the hip into swing (similar to the approach of other researchers [38]). Because there is no time following the exoskeleton recoil in late swing or early stance when the hip would be extended to more than 10 degrees, the device would not require a clutching mechanism to prevent muscles from doing work against the spring to reset the hip configuration for the next stride. We note that it would still be important to perform studies to determine what the optimal slack length/angle of the exoskeleton spring should be for optimal performance. A more sophisticated hip exoskeleton design incorporating active power and control could set the timing of spring engagement during initial stance using a clutch and then implement a position control scheme to control the magnitude of elastic energy stored in a series-elastic actuator throughout the rest of the gait.

In summary, in both passive and active implementations of a hip exoskeleton, the stiffness of the spring or impedance of the motor acting in parallel with the user's muscles will likely significantly impact the performance of the device. We expect that hip exoskeletons with joint stiffness selected according to the user's height and weight could help improve the performance of these devices for subjects walking on level ground. Along these lines, we have taken effort to formulate statistical models to characterizing hip quasi-stiffness during walking as a function of a person's height and weight across walking speed. Our models have different levels of sophistication: the general-form models can estimate the quasi-stiffness over a wide range of gait speeds, and the simpler stature-based models can provide estimates near the preferred gait speed only. We expect that the models developed in this study will provide a reference for designers and clinicians to size the springs of hip exoskeletons without requiring additional subject-specific gait analyses. Future studies should address the practical challenges of applying these models in the design and development of hip exoskeletons for use in both healthy and clinical populations as well as during locomotion tasks other than walking on level ground (e.g. up and downhill, with load carriage). Apart from the field of lower-limb exoskeletons, these models could also be used to improve spring-based modeling and simulations of walking, and the design of bipedal robots.

## Supporting Information

Figure S1 **A schematic model of the support thigh, shank, and foot.** The figure depicts the proximal force and moments of the thigh, shank, and foot segments, and the center of masses (*COM_t_*, *COM_s_*, and *COM_f_*). The ground reaction force and moment are also shown at the center of pressure (*COP*). The figure also shows the orientation angle of the unit vectors of the segments in a sagittal view of the leg on the top right.
(TIF)

Appendix S1 **Inverse dynamics analysis.**
(DOCX)

Table S1 Description of mathematical expressions.
(DOCX)
