# Supplementary figures and images for: Estimation of Quasi-Stiffness of the Human Hip in the Stance Phase of Walking
Source: PLoS One. 2013 Dec 9;8(12):e81841. doi: 10.1371/journal.pone.0081841 (PMC3857237; doi:10.1371/journal.pone.0081841)

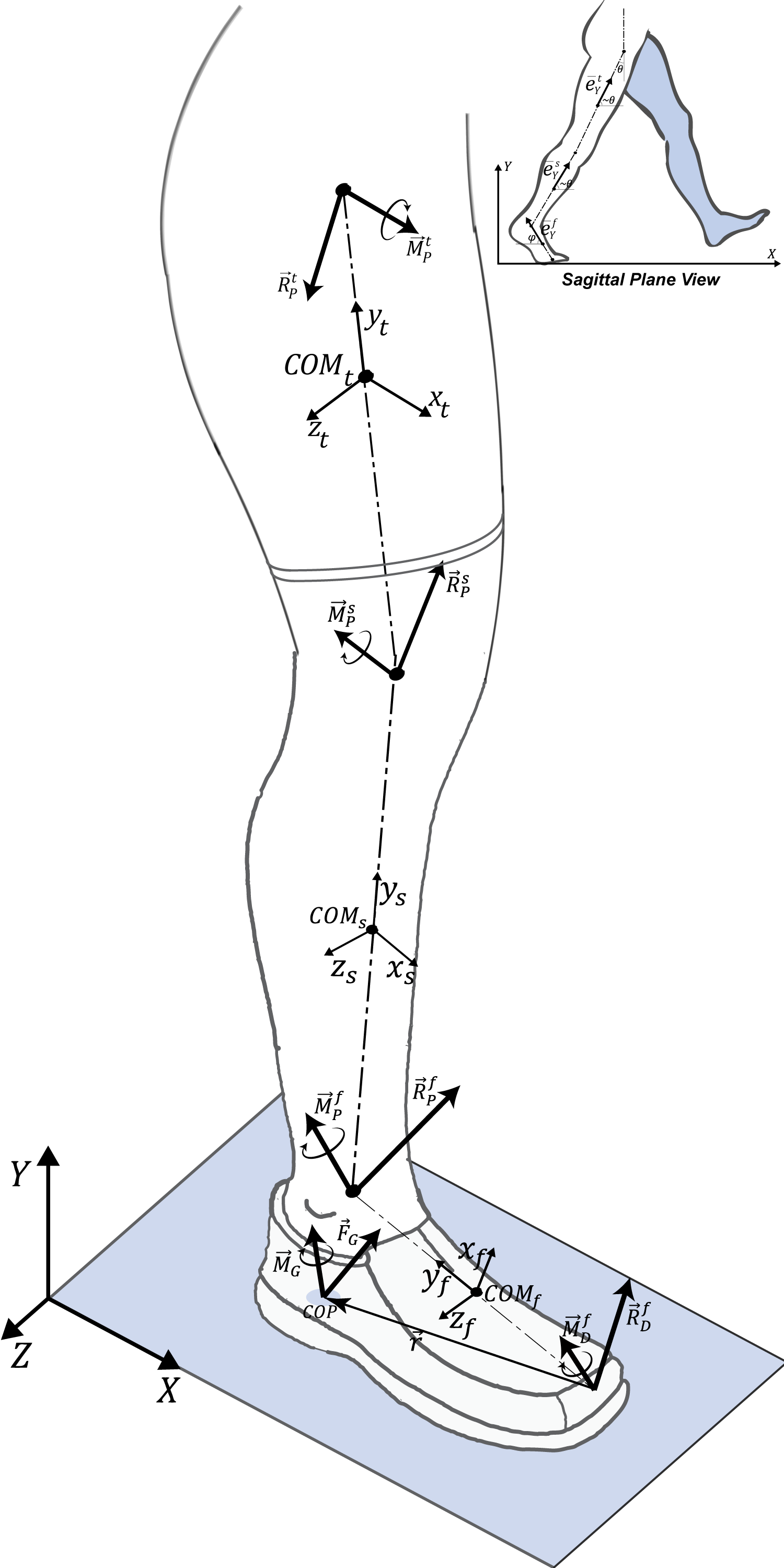

Supplement: Figure S1 — A schematic model of the support thigh, shank, and foot. The figure depicts the proximal force and moments of the thigh, shank, and foot segments, and the center of masses (COMt, COMs, and COMf). The ground reaction force and moment are also shown at the center of pressure (COP). The figure also shows the orientation angle of the unit vectors of the segments in a sagittal view of the leg on the top right. (TIF) [file pone.0081841.s001.tif]
