# Supplementary material for: Estimation of Quasi-Stiffness of the Human Hip in the Stance Phase of Walking
Source: PLoS One. 2013 Dec 9;8(12):e81841. doi: 10.1371/journal.pone.0081841 (PMC3857237; doi:10.1371/journal.pone.0081841)
Supplement: Appendix S1 — Inverse dynamics analysis. (DOCX) [file pone.0081841.s002.docx]

# **APPENDIX: INVERSE DYNAMICS ANALYSIS**

In this section, we derive the equations of the reaction forces and moments for the hip joint. We are mainly interested in the generic expression of the hip moment. Winter presents a detailed explanation of the inverse dynamics analysis used to obtain the lower extremity joints moments and forces [50]. Here, we first obtain the hip reaction forces through the Newtonian equations of motion of thigh with respect to (w.r.t.) the global coordinate frame shown by $X-Y-Z$ in Fig. S1. Next, we derive an expression for the moment of the hip joint w.r.t. the anatomical coordinate frame of the thigh shown by $x_{t}-y_{t}-z_{t}$ in Fig. S1 through the Euler equations and transform it to $X-Y-Z$. The anatomical coordinate frame of the thigh is established by placing $y_{t}$ on the axis connecting the knee to the hip center, $z_{t}$ along with $Z$ and $x_{t}$ along with the cross-product of $y_{t}$ and $z_{t}$. In our previous work [36], we found the following equation for the knee moment:

$$\vec{M}_{P}^{s}=\left\{\vec{M}_{G}-\vec{F}_{G}\times\left(\vec{r}+L_{f}\bar{e}_{Y}^{f}+L_{s}\bar{e}_{Y}^{s}\right)\right\}+$$

$$\left\{\left(m_{f}\vec{a}_{f}+m_{f}g\bar{e}_{Y}\right)\times\left(L_{p}^{f}\bar{e}_{Y}^{f}+L_{s}\bar{e}_{Y}^{s}\right)+\left(m_{s}\vec{a}_{s}+m_{s}g\bar{e}_{Y}\right)\times L_{p}^{s}\bar{e}_{Y}^{s} \right\}+$$

(A-1)

and the following expression for the knee reaction force:

$\vec{R}_{P}^{s}=\vec{F}_{G}+\left(m_{f}\vec{a}_{f}+m_{s}\vec{a}_{s}\right)+\left(m_{f}+m_{s}\right)g\bar{e}_{Y}$ (A-2)

where, $\vec{M}_{G}$ is the ground reaction moment, $\vec{F}_{G}$ground reaction force, $\vec{r}$a vector connecting the center of pressure to the toe, $L_{f}$the length of the foot, $\bar{e}_{Y}^{f}$the unit vector along the anatomical y-axis of the foot, $L_{s}$the length of the shank, and $\bar{e}_{Y}^{s}$the unit vector along the anatomical y-axis of the shank. $m_{f}$is the mass and $\vec{a}_{f}$ the acceleration of the foot, and $m_{s}$is the mass and $\vec{a}_{s}$ the acceleration of the shank. $L_{p}^{f}$is the distance between the foot center of mass and the ankle, and $L_{p}^{s}$ is the distance between the shank center of mass and the knee joint. $\bar{e}_{Y}$is the unit vector along Y-axis and $g$is the acceleration due to gravity. $\left[AG\right]_{s}$ and $\left[AG\right]_{f}$are proper transformations (i.e. reserve inner product and have a determinant of 1) from the anatomical coordinate frames of the shank and foot to the global coordinates system. $\left[I_{s}\right]$ and $\left[I_{f}\right]$are the matrices of moment of inertia for the shank and foot. $\vec{\omega}_{s}$ and $\vec{\omega}_{f}$ are the angular velocities, and the angular accelerations, and $\vec{U}_{s}$ and $\vec{U}_{f}$ are the angular momentums of the shank and foot segments, respectively.

We exploit the expressions for the knee reaction force and moment (i.e. the shank proximal force and moment) as the distal force ($\vec{R}_{D}^{t}$) and moment ($\vec{M}_{D}^{t}$) of the thigh. In other words, w.r.t. $X-Y-Z$ we have:

$\vec{R}_{D}^{t}=-\vec{R}_{P}^{s}$ (A-3-a)

$\vec{M}_{D}^{s}=-\vec{M}_{P}^{s}$ (A-3-b)

which can be transformed to $x_{t}-y_{t}-z_{t}$ using the proper rotation-matrix of $\left[GA\right]_{t}$:

$\vec{R}_{d}^{t}=-\left[GA\right]_{t}\vec{R}_{P}^{s}$ (A-4-a)

$\vec{M}_{d}^{t}=-\left[GA\right]_{t}\vec{M}_{P}^{s}$ (A-4-b)

The hip reaction force is derived using the Newtonian equation of motion for the thigh:

$\sum^{ } \vec{F}_{t}=m_{t}\vec{a}_{t} \therefore\vec{R}_{P}^{t}=\vec{R}_{D}^{t}+m_{t}\vec{a}_{t}+m_{t}g\bar{e}_{Y}$ (A-5)

where,$\vec{F}_{t}$ denotes any force that is applied on the thigh segment, $m_{t}$ is the mass and $\vec{a}_{t}$ is the acceleration of the center of mass ($COM_{t}$) of the thigh. Applying equations (A-3-a) and (A-2) in (A-5), we get:

$\vec{R}_{P}^{t}=\vec{F}_{G}+\left(m_{f}\vec{a}_{f}+m_{s}\vec{a}_{s}+m_{t}\vec{a}_{t}\right)+\left(m_{f}+m_{s}+m_{t}\right)g\bar{e}_{Y}$ (A-6)

which could be transformed to $x_{t}-y_{t}-z_{t}$ as:

$\vec{R}_{p}^{t}=\left[GA\right]_{t}\vec{R}_{P}^{t}$ (A-7)

Now, we employ the Euler equation of motion for the thigh w.r.t. $x_{t}-y_{t}-z_{t}$ to derive the moment of the hip:

(A-8)

where, $\vec{M}_{t}$ denotes any moment that is applied on the thigh. $\left[I_{t}\right]$ is the matrix of moment of inertia, $\vec{\omega}_{t}$ the angular velocity, is the angular acceleration, and $\vec{U}_{t}$ is the angular momentum of the thigh segment. Expanding the left hand side of the above equation results in:

(A-9)

where, $\vec{M}_{p}^{t}$ is the moment at the proximal joint of the thigh (i.e. hip). $\vec{d}_{t}$ is the vector connecting the center of mass of the thigh ($COM_{t}$) to the knee and $\vec{p}_{t}$ the vector connecting $COM_{t}$ to the hip both expressed in $x_{t}-y_{t}-z_{t}$. Inserting the corresponding expressions of each term in (A-18) and transforming the equations to $X-Y-Z$, we conclude:

$$\vec{M}_{P}^{t}=\left\{\vec{M}_{G}-\vec{F}_{G}\times\left(\vec{r}+L_{f}\bar{e}_{Y}^{f}+L_{s}\bar{e}_{Y}^{s}+L_{t}\bar{e}_{Y}^{t}\right)\right\}+$$

$$\left\{\left(m_{f}\vec{a}_{f}+m_{f}g\bar{e}_{Y}\right)\times\left(L_{p}^{f}\bar{e}_{Y}^{f}+L_{s}\bar{e}_{Y}^{s}+L_{t}\bar{e}_{Y}^{t}\right)+\left(m_{s}\vec{a}_{s}+m_{s}g\bar{e}_{Y}\right)\times\left(L_{p}^{s}\bar{e}_{Y}^{s}+L_{t}\bar{e}_{Y}^{t}\right)+\left(m_{t}\vec{a}_{t}+m_{t}g\bar{e}_{Y}\right)\times L_{p}^{t}\bar{e}_{Y}^{t}\right\}$$

(A-10)

where, $L_{t}$ is the length of the thigh segment and $\bar{e}_{Y}^{t}$ is a unit vector along $y_{t}$ expressed w.r.t. $X-Y-Z$, $L_{p}^{t}$ is the distance between $COM_{t}$ and the hip, and $\left[AG\right]_{t}$is a proper transformation from anatomical coordinate frame of the thigh to the global coordinate frame ($\left[AG\right]_{t}=\left[GA\right]_{t}^{-1}$).
