# Supplementary material for: Estimation of Quasi-Stiffness of the Human Hip in the Stance Phase of Walking
Source: PLoS One. 2013 Dec 9;8(12):e81841. doi: 10.1371/journal.pone.0081841 (PMC3857237; doi:10.1371/journal.pone.0081841)
Supplement: Table S1 — Description of mathematical expressions. (DOCX) [file pone.0081841.s003.docx]

**TABLE S1.** Description of the Mathematical Expressions

| **Parameter** | **Description** | **Parameter** | **Description** |
| --- | --- | --- | --- |
| $\boldsymbol{K}_{\boldsymbol{e}}$ | Hip quasi-stiffness in extension stage | ${\vec{\boldsymbol{U}}}_{\boldsymbol{f}}$ | Angular momentum of foot |
| $\boldsymbol{K}_{\boldsymbol{f}}$ | Hip quasi-stiffness in flexion stage | ${\vec{\boldsymbol{U}}}_{\boldsymbol{s}}$ | Angular momentum of shank |
| $\boldsymbol{\theta}_{\boldsymbol{e}}$ | Hip excursion in extension stage | ${\vec{\boldsymbol{U}}}_{\boldsymbol{t}}$ | Angular momentum of thigh |
| $\boldsymbol{\theta}_{\boldsymbol{f}}$ | Hip excursion in flexion stage | $\left[ \boldsymbol{I}_{\boldsymbol{f}} \right]$ | Matrix of moment of inertia of foot |
| $\boldsymbol{W}$ | Body weight | $\left[ \boldsymbol{I}_{\boldsymbol{s}} \right]$ | Matrix of moment of inertia of shank |
| $\boldsymbol{V}$ | Gait speed | $\left[ \boldsymbol{I}_{\boldsymbol{t}} \right]$ | Matrix of moment of inertia of thigh |
| $\boldsymbol{H}$ | Body height | ${\vec{\boldsymbol{M}}}_{\boldsymbol{P}}^{\boldsymbol{t}}$ | Thigh proximal moment in global coordinate system (hip moment) |
| ${\vec{\boldsymbol{M}}}_{\boldsymbol{G}}$ | Ground reaction moment | ${\vec{\boldsymbol{M}}}_{\boldsymbol{D}}^{\boldsymbol{t}}$ | Thigh distal moment in global coordinate system |
| ${\vec{\boldsymbol{F}}}_{\boldsymbol{G}}$ | Ground reaction force | ${\vec{\boldsymbol{M}}}_{\boldsymbol{P}}^{\boldsymbol{s}}$ | Shank proximal moment in global coordinate system (knee moment) |
| $\vec{\boldsymbol{r}}$ | Vector from toe to center of pressure | ${\vec{\boldsymbol{M}}}_{\boldsymbol{D}}^{\boldsymbol{s}}$ | Shank distal moment in global coordinate system |
| $\boldsymbol{L}_{\boldsymbol{f}}$ | Foot length | ${\vec{\boldsymbol{M}}}_{\boldsymbol{P}}^{\boldsymbol{f}}$ | Foot proximal moment in global coordinate system (ankle moment) |
| $\boldsymbol{L}_{\boldsymbol{s}}$ | Shank length | ${\vec{\boldsymbol{M}}}_{\boldsymbol{D}}^{\boldsymbol{f}}$ | Foot distal moment in global coordinate system |
| $\boldsymbol{L}_{\boldsymbol{t}}$ | Thigh length | ${\vec{\boldsymbol{M}}}_{\boldsymbol{p}}^{\boldsymbol{t}}$ | Thigh proximal moment in shank anatomical coordinate system |
| ${\bar{\boldsymbol{e}}}_{\boldsymbol{Y}}^{\boldsymbol{f}}$ | Unit vector along foot segment | ${\vec{\boldsymbol{M}}}_{\boldsymbol{d}}^{\boldsymbol{t}}$ | Thigh distal moment in shank anatomical coordinate system |
| ${\bar{\boldsymbol{e}}}_{\boldsymbol{Y}}^{\boldsymbol{s}}$ | Unit vector along shank segment | ${\vec{\boldsymbol{M}}}_{\boldsymbol{p}}^{\boldsymbol{s}}$ | Shank proximal moment in shank anatomical coordinate system |
| ${\bar{\boldsymbol{e}}}_{\boldsymbol{Y}}^{\boldsymbol{t}}$ | Unit vector along thigh segment | ${\vec{\boldsymbol{M}}}_{\boldsymbol{d}}^{\boldsymbol{s}}$ | Shank distal moment in shank anatomical coordinate system |
| $\boldsymbol{m}_{\boldsymbol{f}}$ | Foot mass | ${\vec{\boldsymbol{M}}}_{\boldsymbol{p}}^{\boldsymbol{f}}$ | Foot proximal moment in shank anatomical coordinate system |
| $\boldsymbol{m}_{\boldsymbol{s}}$ | Shank mass | ${\vec{\boldsymbol{M}}}_{\boldsymbol{d}}^{\boldsymbol{f}}$ | Foot distal moment in shank anatomical coordinate system |
| $\boldsymbol{m}_{\boldsymbol{t}}$ | Thigh mass | ${\vec{\boldsymbol{R}}}_{\boldsymbol{P}}^{\boldsymbol{t}}$ | Thigh proximal force in global coordinate system |
| ${\vec{\boldsymbol{a}}}_{\boldsymbol{f}}$ | Foot acceleration | ${\vec{\boldsymbol{R}}}_{\boldsymbol{D}}^{\boldsymbol{t}}$ | Thigh distal force in global coordinate system |
| ${\vec{\boldsymbol{a}}}_{\boldsymbol{s}}$ | Shank acceleration | ${\vec{\boldsymbol{R}}}_{\boldsymbol{P}}^{\boldsymbol{s}}$ | Shank proximal force in global coordinate system |
| ${\vec{\boldsymbol{a}}}_{\boldsymbol{t}}$ | Thigh acceleration | ${\vec{\boldsymbol{R}}}_{\boldsymbol{D}}^{\boldsymbol{s}}$ | Shank distal force in global coordinate system |
| $\boldsymbol{g}$ | Magnitude of acceleration due to gravity | ${\vec{\boldsymbol{R}}}_{\boldsymbol{P}}^{\boldsymbol{f}}$ | Foot proximal force in global coordinate system |
| $\boldsymbol{L}_{\boldsymbol{p}}^{\boldsymbol{f}}$ | Distance between center of mass of foot to ankle | ${\vec{\boldsymbol{R}}}_{\boldsymbol{D}}^{\boldsymbol{f}}$ | Foot distal force in global coordinate system |
| $\boldsymbol{L}_{\boldsymbol{p}}^{\boldsymbol{s}}$ | Distance between center of mass of shank to knee | ${\vec{\boldsymbol{R}}}_{\boldsymbol{p}}^{\boldsymbol{t}}$ | Thigh proximal force in shank anatomical coordinate system |
| $\boldsymbol{L}_{\boldsymbol{p}}^{\boldsymbol{t}}$ | Distance between center of mass of thigh to hip | ${\vec{\boldsymbol{R}}}_{\boldsymbol{d}}^{\boldsymbol{t}}$ | Thigh distal force in shank anatomical coordinate system |
| $\left[ \boldsymbol{AG} \right]_{\boldsymbol{t}}$ | Transformation matrix from anatomical coordinate frame of thigh to global coordinate frame | ${\vec{\boldsymbol{R}}}_{\boldsymbol{p}}^{\boldsymbol{s}}$ | Shank proximal force in shank anatomical coordinate system |
| $\left[ \boldsymbol{GA} \right]_{\boldsymbol{t}}$ | Transformation matrix from global coordinate frame to anatomical coordinate frame of thigh | ${\vec{\boldsymbol{R}}}_{\boldsymbol{d}}^{\boldsymbol{s}}$ | Shank distal force in shank anatomical coordinate system |
| $\left[ \boldsymbol{AG} \right]_{\boldsymbol{s}}$ | Transformation matrix from anatomical coordinate frame of shank to global coordinate frame | ${\vec{\boldsymbol{R}}}_{\boldsymbol{p}}^{\boldsymbol{f}}$ | Shank proximal force in shank anatomical coordinate system |
| $\left[ \boldsymbol{GA} \right]_{\boldsymbol{s}}$ | Transformation matrix from global coordinate frame to anatomical coordinate frame of shank | ${\vec{\boldsymbol{R}}}_{\boldsymbol{d}}^{\boldsymbol{f}}$ | Shank distal force in shank anatomical coordinate system |
| $\left[ \boldsymbol{AG} \right]_{\boldsymbol{f}}$ | Transformation matrix from anatomical coordinate frame of foot to global coordinate frame | $\boldsymbol{X-Y-Z}$ | Global coordinate system |
| $\left[ \boldsymbol{GA} \right]_{\boldsymbol{f}}$ | Transformation matrix from global coordinate frame to anatomical coordinate frame of foot | $\boldsymbol{x}_{\boldsymbol{t}}\boldsymbol{-}\boldsymbol{y}_{\boldsymbol{t}}\boldsymbol{-}\boldsymbol{z}_{\boldsymbol{t}}$ | Anatomical coordinate system of thigh |
| ${\vec{\boldsymbol{\omega}}}_{\boldsymbol{t}}$ | Angular velocity of thigh | $\boldsymbol{x}_{\boldsymbol{s}}\boldsymbol{-}\boldsymbol{y}_{\boldsymbol{s}}\boldsymbol{-}\boldsymbol{z}_{\boldsymbol{s}}$ | Anatomical coordinate system of shank |
| ${\vec{\dot{\boldsymbol{\omega}}}}_{\boldsymbol{t}}$ | Angular acceleration of thigh | $\boldsymbol{x}_{\boldsymbol{f}}\boldsymbol{-}\boldsymbol{y}_{\boldsymbol{f}}\boldsymbol{-}\boldsymbol{z}_{\boldsymbol{f}}$ | Anatomical coordinate system of foot |
| ${\vec{\boldsymbol{\omega}}}_{\boldsymbol{s}}$ | Angular velocity of shank | $\boldsymbol{M}_{\boldsymbol{H}}^{\boldsymbol{Z}}$ | Hip moment on the sagittal plane |
| ${\vec{\dot{\boldsymbol{\omega}}}}_{\boldsymbol{s}}$ | Angular acceleration of shank | ${\vec{\boldsymbol{d}}}_{\boldsymbol{f}}$ | Vector connecting center of mass of foot to toe |
| ${\vec{\boldsymbol{\omega}}}_{\boldsymbol{f}}$ | Angular velocity of foot | ${\vec{\boldsymbol{p}}}_{\boldsymbol{f}}$ | Vector connecting center of mass of foot to ankle |
| ${\vec{\dot{\boldsymbol{\omega}}}}_{\boldsymbol{f}}$ | Angular acceleration of foot | ${\vec{\boldsymbol{d}}}_{\boldsymbol{s}}$ | Vector connecting center of mass of shank to ankle |
| ${\boldsymbol{C}\boldsymbol{OM}}_{\boldsymbol{t}}$ | Center of mass of thigh | ${\vec{\boldsymbol{p}}}_{\boldsymbol{s}}$ | Vector connecting center of mass of shank to knee |
| $\boldsymbol{COM}_{\boldsymbol{s}}$ | Center of mass of shank | ${\vec{\boldsymbol{d}}}_{\boldsymbol{t}}$ | Vector connecting center of mass of thigh to knee |
| $\boldsymbol{COM}_{\boldsymbol{f}}$ | Center of mass of foot | ${\vec{\boldsymbol{p}}}_{\boldsymbol{t}}$ | Vector connecting center of mass of thigh to hip |
| ${\vec{\boldsymbol{M}}}_{\boldsymbol{t}}$ | Any moment applied on thigh | ${\vec{\boldsymbol{F}}}_{\boldsymbol{t}}$ | Any force applied on thigh |
| ${\vec{\boldsymbol{M}}}_{\boldsymbol{s}}$ | Any moment applied on shank | ${\vec{\boldsymbol{F}}}_{\boldsymbol{s}}$ | Any force applied on shank |
| ${\vec{\boldsymbol{M}}}_{\boldsymbol{f}}$ | Any moment applied on foot | ${\vec{\boldsymbol{F}}}_{\boldsymbol{f}}$ | Any force applied on foot |
| $\boldsymbol{Fr}$ | Froude number for walking | ${\bar{\boldsymbol{e}}}_{\boldsymbol{Y}}$ | Unit vector vertical to the ground and along $Y$ |
